# Exploring factors that influence the spread and sustainability of a dysphagia innovation: an instrumental case study

**DOI:** 10.1186/s12913-016-1653-6

**Published:** 2016-08-18

**Authors:** Irene Ilott, Kate Gerrish, Sabrina A. Eltringham, Carolyn Taylor, Sue Pownall

**Affiliations:** 1 Formerly Knowledge Translation Project Lead with the NIHR CLAHRC SY, Sheffield, UK; 2 School of Nursing and Midwifery University of Sheffield and Sheffield Teaching Hospitals NHS Foundation Trust, NIHR CLAHRC Yorkshire and Humber, Sheffield, UK; 3 Sheffield Teaching Hospitals NHS Foundation Trust, D Floor Research, Royal Hallamshire Hospital, Glossop Road, Sheffield, S10 2JF UK; 4 Sheffield Teaching Hospitals NHS Foundation Trust, Sheffield, UK; 5 Dietetic Department, Sheffield Teaching Hospital NHS Foundation Trust, Northern General Hospital, Herries Road, Sheffield, S5 7AU UK

**Keywords:** Spread, Sustainability, Dysphagia, Patient safety, Instrumental case study, Small theory

## Abstract

**Background:**

Swallowing difficulties challenge patient safety due to the increased risk of malnutrition, dehydration and aspiration pneumonia. A theoretically driven study was undertaken to examine the spread and sustainability of a locally developed innovation that involved using the Inter-Professional Dysphagia Framework to structure education for the workforce. A conceptual framework with 3 spread strategies (hierarchical control, participatory adaptation and facilitated evolution) was blended with a processual approach to sustaining organisational change. The aim was to understand the processes, mechanism and outcomes associated with the spread and sustainability of this safety initiative.

**Methods:**

An instrumental case study, prospectively tracked a dysphagia innovation for 34 months (April 2011 to January 2014) in a large health care organisation in England. A train-the-trainer intervention (as participatory adaptation) was deployed on care pathways for stroke and fractured neck of femur. Data were collected at the organisational and clinical level through interviews (*n =* 30) and document review. The coding frame combined the processual approach with the spread mechanisms. Pre-determined outcomes included the number of staff trained about dysphagia and impact related to changes in practice.

**Results:**

The features and processes associated with hierarchical control and participatory adaptation were identified. Leadership, critical junctures, temporality and making the innovation routine were aspects of hierarchical control. Participatory adaptation was evident on the care pathways through stakeholder responses, workload and resource pressures. Six of the 25 ward based trainers cascaded the dysphagia training. The expected outcomes were achieved when the top-down mandate (hierarchical control) was supplemented by local engagement and support (participatory adaptation).

**Conclusions:**

Frameworks for spread and sustainability were combined to create a ‘small theory’ that described the interventions, the processes and desired outcomes a priori. This novel methodological approach confirmed what is known about spread and sustainability, highlighted the particularity of change and offered new insights into the factors associated with hierarchical control and participatory adaptation. The findings illustrate the dualities of organisational change as universal and context specific; as particular and amendable to theoretical generalisation. Appreciating these dualities may contribute to understanding why many innovations fail to become routine.

**Electronic supplementary material:**

The online version of this article (doi:10.1186/s12913-016-1653-6) contains supplementary material, which is available to authorized users.

## Background

Spread and sustainability are critical to patient safety initiatives. Patient safety problems exist in health care systems around the world [1]. The cost of preventable, adverse events to the National Health Service in England has been estimated at between £1 billion and £2.5 billion each year [2]. Swallowing difficulties are a patient safety concern because of the increased risk of malnutrition, dehydration and aspiration pneumonia which can lead to death [3]. Dysphagia is also linked with poor outcomes including disability, and longer hospital stay [4]. A number of treatments for dysphagia have been studied, which aim to improve swallowing and to reduce the risk of the person developing aspiration pneumonia. A Cochrane systematic review of interventions for dysphagia in stroke concluded there was insufficient evidence on whether swallowing therapy affects death, disability or dependency [5]. Over the last decade dysphagia has been the subject of national patient safety alerts in England [6, 7].

Although spread and sustainability are ill-defined, under theorised concepts [8–12] they are attracting increasing attention. Spread is associated with an innovation spreading along a care pathway or across the health care system. Sustainability is ‘the process through which new working methods, performance enhancements, and continuous improvements are maintained for a period appropriate to a given context’ [8], p.231. Both concepts have been investigated through primary and secondary research [13–17] including an evidence scan of spread [18] and a concept analysis of sustainability [12].

Our research comprised an instrumental case study [19] whereby a specific instance (uptake of dysphagia recommendations) was examined to learn about spread and sustainability. The details were published in the protocol [20]. An earlier study undertaken by the research team promoted dysphagia as a patient safety issue [21] and recommended using the Inter-Professional Dysphagia Framework [22] to structure education for the workforce. The Inter-Professional Dysphagia Framework (IPDF) was developed by several professional bodies to offer a consistent approach to competency development for knowledge and skills in dysphagia in the United Kingdom. It comprises 5 levels, from Awareness which introduces the risks of dysphagia, through to Consultant Dysphagia Practitioners who undertake specialist investigations, manage complex cases and contribute to research. We chose to focus on the second level: Assistant Dysphagia Practitioner because it covers the knowledge and skills needed to support safe swallowing and applies to anyone who assists patients to eat and drink. Information about how we applied the Framework is presented in the South Yorkshire Dysphagia Toolkit [23] at http://www.dysphagiatoolkit.nihr.ac.uk/.

Two theoretical frameworks were used to examine the spread and sustainability of this patient safety initiative about dysphagia. These were Ovretveit’s [10] three spread strategies. These are hierarchical control which refers to a top-down ‘push’ with senior level decision making and operational staff held to account for making the change. Participatory adaptation is where decision making is more collaborative, focusing on principles and examples; and support is provided for local adaptation alongside regular feedback from experts. Facilitated evolution emphasizes capacity building, facilitation, making resources available to solve the problem, rather than prescribing change and the adoption process. These spread mechanisms were combined with the processual perspective described in a literature review about sustaining organisational change [24]. This approach focusses ‘on the substance and process of change in context’ [24], p.202. It includes critical junctures or factors triggering action, the decisions of key stakeholders, and the timing of events [8].

Both frameworks are descriptive [25] offering a ‘small’ theory [26] or conceptual lens to investigate the research aims. A small theory makes explicit the interventions, the processes and desired outcomes in advance, in an attempt to strengthen causal interpretation from specific studies [26]. The research aimed to scrutinise the processes and outcomes associated with the diffusion of a locally developed innovation about dysphagia; and also to identify the factors that influence the spread and sustainability of the dysphagia innovation.

The paper describes this novel, theoretically driven approach and reports findings about using the IPDF for workforce development. As far as we are aware, this is the first time that the Framework has been applied across a health care organisation. The study illustrates the dualities of organisational change as context specific and universal; particular and amendable to theoretical generalisation. Exploring these dualities may contribute to understanding the variance in the uptake of research findings into health care practice.

## Methods

### Design

A prospective, longitudinal design tracked the spread and sustainability of the dysphagia recommendation from April 2011 to January 2014 at organisational and clinical levels. The recommendation was to use the Inter-Professional Dysphagia Framework to structure education for the whole workforce. The organisation was a publically funded health care facility in England (NHS Trust) which was the setting for the original study and was receptive to further research [20]. It employed around 15,500 staff serving in excess of 1 million patients each year. The clinical level included hospital wards and a community unit providing acute care and rehabilitation for patients on the care pathways for stroke and fractured neck of femur. Dysphagia is an anticipated problem post stroke [3, 4], whereas it is less so for frail, older people with a hip fracture even though the prevalence in the elderly population ranges from 7 to 22 % [27]. The care pathways comprised 7 wards, 5 in hospital and 2 in the community (see Table 1).

**Table 1:** Settings: number of beds and range in staff numbers on the stroke and fractured neck of femur care pathways

Characteristic	Stroke care pathway		Fractured neck of femur care pathway			
	*Acute Stroke Unit*	*Rehab. in community*	Post-op.	Post-op.	Rehab.	Rehab. in community
Number of beds	*56*	*31* ^*a*^	34	34	28	31^a^
Number of staff	*60-70* ^*b*^	*50* ^*c*^	39–41	36–38	39	50^c^

^a^Community unit with 31 beds for stroke rehabilitation and frail elderly orthopaedic rehabilitation

^b^Stroke service staff rotate between the hyper acute and acute wards

^c^Rehabilitation in community unit staff rotate between both wards and some work in the community

### Interventions

The interventions were categorised according to Ovretveit’s framework [10]. Hierarchical control was tracked through the formalisation of the IPDF in organisation wide education policies. Participatory adaptation comprised a train-the-trainer intervention to prepare a cadre of ward based trainers to deliver Awareness and Assistant Dysphagia Practitioner competence [22]. This lasted from January to October 2013 and comprised a 3 hour session with a speech and language therapist (SLT), a Dysphagia Toolkit with teaching resources and information, three e-learning programmes which contained the essential knowledge, out-reach visits by the SLT, written and verbal feedback; and additional activities in response to requests (see Table 2). The learning effect of the train-the-trainer intervention was evaluated using a postal survey of dysphagia knowledge and attitudes, and a structured observation of staff adherence to patient-specific, dysphagia management recommendations at mealtimes on the care pathways. Facilitated evolution involved putting the Dysphagia Toolkit on the organisation’s intranet. Each intervention and the evaluation tools are described in Additional file 1.

**Table 2:** Participatory Adaptation: description of the train-the-trainer intervention on the stroke and fractured neck of femur care pathways

	*Stroke care pathway*		Fractured neck of femur care pathway^a^			
	*Acute Stroke*	*Rehab.* ^*c*^ *in community*	Post-op^b^ ward	Post-op.^b^ ward	Rehab.^c^ ward	Rehab.^c^ in community
Staff trained as local Trainers						
Number of staff	*3*	*9* ^*d*^	5	4	4	9^d^
Designation of the trainers	*2 RNs* *1 OT*	*2 RNs* *5 CSWs* *2 CMs*	2 RNs 2 CSWs 1H	2 RNs 2 CSWs	2 RNs 2 CSWs	2 RNs 5 CSWs 2 CMs
Follow-up support provided to the local Trainers between January–October 2013						
Outreach: number of drop-in sessions	*4*	*1* ^*d*^	8	8	8	1^d^
Feedback: verbal and written reports at half-way and end	*√*	*√*	√	√	√	√
Adaptation: additional activities	*Provided staff notices about mixed diet consistencies*	*Joint teaching with the catering team*	Prepared resource box with equipment to deliver the training Co-led a funding bid to buy specialist dysphagia and adapted crockery/cutlery			Joint teaching with the catering team
Training delivered by the trainers at Awareness and Assistant Dysphagia Practitioner levels						
Formal: staff trained by ward based Trainers at 6 months	*20–30* ^*e*^	*2* ^*d*^	0	15 (Awareness level)	0	2^d^
Formal: number of staff trained by 3 Education Leads^a^ at 10 months	*-*	*-*	Surgical Services Directorate 176 RNs 97 CSWs			-
Informal: sharing knowledge, self-report and testimony from others	*√*	*√*	√	√	√	√

^a^Three Education Leads for the Surgical Services volunteered to be trained as Dysphagia Trainers. These were in addition to the ward based Trainers

^b^Post-operative ward. ^c^Rehabilitation. ^d^Community rehabilitation unit staff rotates between the care pathways

Abbreviations: *RN* registered nurse, *OT* occupational therapist, *CSW* clinical support worker, *H* housekeeper and, *CM* catering manager. ^e^Estimated number (range) of stroke service staff trained by the dysphagia trainer

### Participants

Interviewees were purposively sampled according to their roles at organisational and clinical levels. There were senior managers with an organisation-wide remit. These are referred to as Education Strategic Leads (ESL) and Professional Strategic Leads (PSL). On the care pathways, there were Clinical Leads (CL), Education Leads (EL) and Trainers (T) who completed the train-the-trainer course.

### Data collection methods

The methods were interviews and document review. The semi-structured individual and group interviews covered understanding of the dysphagia recommendations, experience of the interventions and examples of change in clinical practice (see Additional file 2). Thirty participants were interviewed between July and October 2013 by II, with 1 exception: an external expert associated with the IPDF was interviewed by SAE. The mean duration of the 17 individual interviews was 38 min. The 5 group interviews with 13 participants lasted a mean of 39 min. All the interviews were recorded and transcribed, except for 1 group interview when a participant declined and contemporaneous notes were taken.

Three documents were reviewed. These were a contemporaneous ethnographic field work journal [28] kept by 2 researchers (II, SAE). The journal had 209 entries between April 2011 and January 2014. Secondly, organisation-wide Education and Training Policies published on the intranet. Finally, the Essential Skills Log Book, containing competences to be demonstrated by newly qualified Registered Nurses (RNs) within the first 6 months.

### Analysis

The Framework Approach [29] was used to analyse the interview transcripts and journal. Textual data from the transcripts was managed using QSR NVivo 8. The data from each care pathway were brought together from the outset using our ‘small theory’. Text was coded according to predetermined processes of sustainability [8]; the mechanisms of spread [10]; and the outcomes were references to the IPDF in policy documents and the number of staff trained about dysphagia. Indicators of impact about changes in practice were left open. Next, the coded extracts from the journal and transcripts were combined to permit triangulation and create the timelines. Triangulation involved synthesis and corroboration between the interviews and field work journal to gain a more complete picture [30].

The first author (II), with over 20 years’ experience of qualitative research, took the main role in analysis. Two people (CT, SAE) followed a data thread [30] by independently selecting a process (such as critical junctures), scrutinising the coded data, reviewing the analysis and interpretation. Member checking was carried out by the SLT Lead for Dysphagia (SP) to ensure the credibility of interpretation from the organisational perspective. The findings were considered by the whole team and agreement was reached on the mechanisms, processes and outcomes. An audit trail was maintained throughout.

## Results

### Demographic details

The majority of participants were front-line staff working on the care pathways (22/30), seven had an organisation wide role and one was an external expert. Twelve of the 25 Trainers were interviewed, some of whom were also Clinical or Education Leads. Most were female (73 % *n =* 22) and were RNs (56 % *n =* 17) (Table 3). Although two thirds (66 % *n =* 20) had been in their current post for less than 5 years, many had worked for the organisation for more than a decade.

**Table 3:** Demographic details of the 30 interviewees

	*Organisation wide*	Stroke care pathway	*Fractured neck of femur care pathway*	Rehabilitation facility in the community (both care pathways)
Role	*4 Education Strategic Leads* *3 Professional Strategic Leads* *1 external*	2 Trainers 2 Clinical Leads 1 Education Lead	*8 Trainers* *3 Education Leads* *1 Clinical Lead*	2 Trainers 3 Clinical Leads
Profession	*4 RNs* *2 SLTs* *2 Others*	4 RNs 1 OT	*7 RNs* *4 CSWs* *1 Other*	2 RNs 1 SLT 1 Dietitian 1 Other
Gender	*6 Female* *2 Male*	4 Female 1 Male	*8 Female* *4 Male*	4 Female 1 Male
Tenure in current post	*˂ 12 months: 1* *1*–*5 years: 3* *6*–*10 years: 2* *˃11 years: 1* *NR: 1* ^*a*^	˂ 12 months:1 1–5 years: 3 ˃11 years: 1	*˂ 12 months:1* *1*–*5 years: 7* *6*–*10 years: 2* *˃11 years: 2*	1–5 years: 4 ˃11 years: 1

Abbreviations: *RN* Registered Nurse, *SLT* Speech and Language Therapist, *OT* Occupational Therapist, *CSW* Clinical Support Worker, Others: Housekeeper, Catering Manager and Trainers. ^a^Not recorded

### Processual approach

#### Stakeholder response

Key stakeholders were senior managers and clinicians with the power to approve the recommendations and then facilitate implementation within their area of responsibility. The importance of having an executive level sponsor was noted by 3 senior managers. An Education Strategic Lead suggested this was ‘helpful when you are trying to do a Trust wide change … because, if they feel confident in it, it will makes sense for everyone else and you need consistency’ (ESL2).

The message that dysphagia was a patient safety issue that required staff training was uncontested. However, three concerns threatened the acceptance of the recommendations at the beginning. These were the generality of the recommendations, whether there would be resources for implementation, and the mismatch between a national dysphagia competency framework and levels of staff development in the local education policy. These points were raised by committees with decision making powers and individuals. For example, the 2011 field notes refer to an email correspondence with a clinical lead about the need for specifics about ‘what should be done.’ These concerns were addressed through the task and finish groups, described in Additional file 1. The mismatch in language was remembered 2 years later: ‘You used a different language from what the Trust was used to, about the levels, for the training needs analysis and the mandatory strategy. I think that was the challenge: trying to interpret, translate into (our) language, words and frameworks’ (ESL2).

**Fig. 1:**
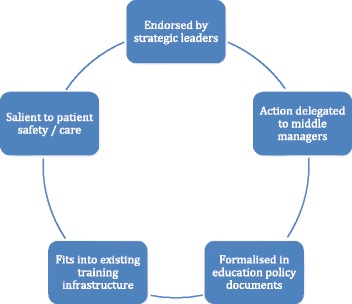
Features of hierarchical control

Stakeholders remained positive throughout, using their authority to secure support and action within their team, a feature of hierarchical control (Fig. 1). For example, ‘Once I realise something is important and we need to sort it out … I think the manager’s job is to create the environment whereby hopefully the work is a success’ (PSL 3). The reasons interviewees gave for being supportive related to their professional experience, concerns about patient safety and agreement with the message that ‘dysphagia is everyone’s business’. Typical examples were ‘I can think of patients I’ve nursed, that if I had known this stuff, it would have made a difference’ (EL&T2) and ‘this is very important training which should involve anyone who is involved with a patient’ (T5).

#### Critical junctures

A critical juncture was a moment, when a decision or some factor shaped what happened next, either directly or indirectly. The first-an unexpected example of sustainability-was the starting point for this study. The 2011 field notes contain a telephone conversation between a researcher and a clinical leader on the stroke care pathway who described a patient safety action (removing thickener from the bedside to avoid inappropriate use) they had instituted after participating in the first study [21].

**Fig. 2:**
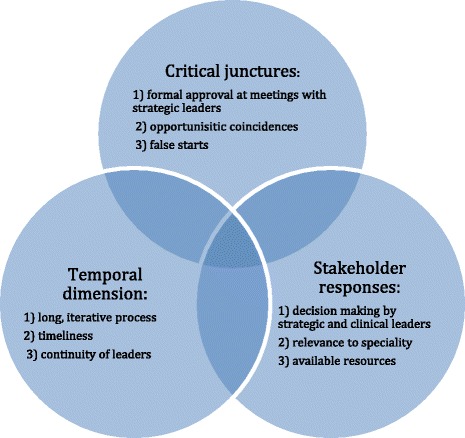
Processes associated with hierarchical control

Critical junctures were associated with hierarchical control (Fig. 2). They included formal meetings with organisational and clinical leaders which were characterised by persuasion about the need for workforce development about dysphagia. The field notes record ten such meetings (4 in 2011, 4 in 2012, 1 in 2013 and 1 in 2014). Stakeholder responses ranged from endorsement of the Dysphagia Framework to uncertainty which required follow-up work about the relevance of the recommendations for the allied health professions.

**Fig. 3:**
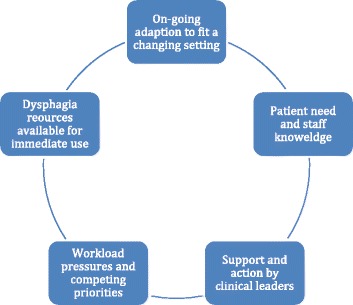
Features of participatory adaptation

**Fig. 4:**
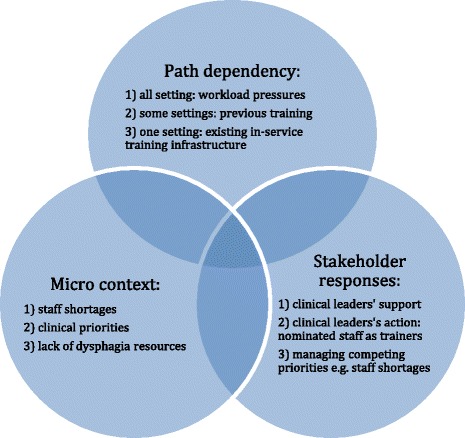
Processes associated participatory adaptation

Some critical junctures seemed opportunistic because they were timely and linked to personal relationships, illustrating a combination of hierarchical control and participatory adaptation (Figs. 1, 2, 3 and 4). Two occurred at informal meetings, intended to update colleagues about progress and were documented in the 2012 field notes. The first example relates to adding the dysphagia competences to the preceptorship scheme for new qualified RNs. The conversation coincided with the preparation of the new Essential Skills Log Book and an Education Strategic Lead liked the idea because staff nurses and their preceptors would gain the competences simultaneously. The second instance comes from the fractured neck of femur care pathway. An education lead recalled that our meeting overlapped with a mandate from the manager to address dysphagia, saying ‘just when the issue was raised again … it all came together … We had a solution to how we could get screeners and a solution to how we could implement the general awareness about dysphagia and patient safety’ (EL&T1).

Four moments were identified as critical junctures at the time but the expected action or decision did not happen. All related to hierarchical control. There was the inaction of senior managers or organisation-wide committees when they did not institute any activity, training or support to spread the recommendations to a wider group of staff. A notable false start was when none of the preceptors (senior RNs mentoring newly qualified nurses) followed up our offer of help when the dysphagia competences were added to the Essential Skills Log Book.

#### Temporal dimension

Tables 4, 5 and 6 show the time line for hierarchical control, participatory adaptation and the research. It illustrates circularity with the research starting and ending with targeted dissemination of the findings from the first [21], and this study.

**Table 4:** 2011 Timeline: Activities associated with the mechanism of hierarchical control and the start of the research

	April 2011	May-August 2011	September 2011	October 2011	November 2011	December 2011
Hierarchical control	Findings from first study [21] presented to the Stroke Management Team	Targeted dissemination of findings to hospital and community staff	Findings presented to the Nutrition Group	Findings presented to the Nurse Leaders	Findings presented to the Stroke Management Team	Task and finish group set up by the Nutrition Group
Research	-	-	-	^*a*^ *Starting point: sustainability in stroke unit*		*Exploring research ideas about spread and sustainability*

^*a*^Critical juncture

**Table 5:** 2012 Timeline: Activities associated with hierarchical control and stages of the research

	January 2012	February 2012	March 2012	April 2012	May 2012	June 2012
Hierarchical control	Findings presented to the Allied Health Professionals Quality Group	-	^a^Nutrition Group approved the recommendations from the first study	Stroke unit task and finish group meeting	-	-
Participatory adaptation	*-*	-	-	-	-	-
*Research*	*Reviewing literature*	*Reviewing literature*	-	*Developing proposal*	*Developing proposal*	*Developing proposal*
	July 2012	August 2012	September 2012	October 2012	November 2012	December 2012
Hierarchical control	^a^Dysphagia included in the Essential Skills Log Book for newly qualified Registered Nurses (RNs)	^a^ Nurse Leaders approved the recommendations about using the Dysphagia Framework	-	Stroke unit task and finish group meeting	^a^Directive to include in induction and training for nursing staff in Surgical Services Directorate	-
Participatory adaptation	-	-	-	-	-	-
*Research*	*Ethical approval*	*-*	*Obtaining research governance*	*Research governance approval*	*Data collection: mealtime observations*	*Mealtime observations and knowledge & attitude survey*
				*Research set-up meetings*		

^a^Critical juncture

**Table 6:** 2013–2014 Timeline: Activities associated with the mechanisms of hierarchical control, participatory adaptation and the stages of the research

	January 2013	February 2013	March 2013	April 2013	May 2013	June 2013
Hierarchical control	Dysphagia in local induction and annual training programme for nursing staff in surgical services	-	Dysphagia master classes in induction for new qualified Registered Nurses	-	-	-
Participatory adaptation	*Train-the-trainer sessions; outreach support visits*	*Train-the-trainer sessions; outreach support visits; providing staff notices about mixed consistencies (acute stroke)*	*Train-the-trainer sessions; outreach support visits*	*Feedback meetings and written report to both care pathways; outreach support visits*	*Feedback meetings; outreach support visits; providing training resource materials (post-op fractured neck of femur NoF)*	*Outreach support visit co-teaching (community rehabilitation unit)*
Research	Mealtime observations	Mealtime observations	-	-	Mealtime observations	Mealtime observations
	July 2013	August 2013	September 2013	October 2013	November–December 2013	January 2014
Hierarchical control	*Dysphagia master classes in induction for new RNs*	*-*	*-*	*Dysphagia master classes in induction for new RNs*	*Findings and preliminary recommendations agreed by research management group, including patient /public members*	*Findings presented to the Nutrition Group. Approved recommendations.*
				^*a*^ *Dysphagia in annual up-date training for OTs and PTs*		
Participatory adaptation	-	-	Extra train-the-trainer session for a new trainer (post-op NoF)	Written feedback; co-led funding bid for ward specialist equipment (NoF)	Extra train-the-trainer session for 2 new trainers in community setting	Train-the-trainer session requested by new staff given this role (stroke)
*Research*	*Mealtime observations* *Knowledge & attitude survey* *Interviews*	*Interviews* *Data analysis*	*Interviews* *Data analysis* *Team meeting: headline findings*	*Interviews* *Data synthesis about spread and sustainability*	*Member checking with the research management group*	*Executive summary sent to all participants*

^a^Critical juncture

The recommendations were described as timely for individuals, for clinical and organisational reasons. A trainer commented ‘Yes, for me as a nurse on that ward … I did feel that it was timely’ (T9) because dysphagia had become ‘very common’ on the fractured neck of femur care pathway. A speech and language therapist (CL6) noted that the training coincided with the launch of new national dysphagia diet food texture descriptors [31]. At the organisational level, the Trust was a partner in a 5 year programme of applied research about getting research into practice [32] which meant that resources were available to undertake the work (PSL1). Participants also acknowledged that change takes time.

#### Context

Macro level, external policy influences were described by 10 professional, clinical and education leads, four of whom had an organisation wide remit. Influences included the Francis Inquiry Report [33] that detailed the failings in care in another organisation in England; the increased emphasis on nutrition and staff training. Others were indirectly related to dysphagia, including government performance targets, changes in policy for support worker training and budget constraints.

A variety of meso level factors in the health care organisation helped or hindered the uptake of the recommendations according to 7 professional, clinical and education leaders. Some influences were ongoing, others were anticipated and a few were particular to the organisation. Winter pressures and staff shortages featured in the field notes fives time between January and April 2013, when the train-the-trainer intervention started. A Professional Strategic Lead observed that ‘there is always churn in the system’ (PSL 2) referring to staff resignations, ill health and maternity leave. Organisational change included integrating acute and community services into one organisation and relocating stroke wards to provide the stroke service on one hospital site. The continuity of leaders with an interest in dysphagia and local activities promoting nutrition and hydration were considered helpful.

Factors at the micro level of the care pathways directly affected the capacity of front-line staff to deliver dysphagia training. The 2012 field notes about the research set-up meetings record the goodwill of the clinical leaders to the train-the-trainer intervention; as well as a potential problem: releasing staff to attend the training due to staff shortages and workload. The 2013 field notes about the outreach visits, supporting trainers in their new role, show that most were ‘struggling’ to start cascading the training to their ward colleagues within the first month. This was because of staff shortages, clinical priorities, lack of time during a busy 12 h shift, and the availability of computers with internet access for the e-learning programmes. These difficulties were raised as part of the feedback with the clinical leaders at the mid-way and final meetings.

#### Path dependency

The processual approach assumes that implementation is dependent on prior events. We monitored the concurrent events to identify influences on participatory adaptation and these were checked during the interviews.

There were notable similarities between the care pathways, particularly the increasing incidence of dysphagia and workload pressures. All settings experienced a turnover of trainers, with 20 % (5/25) leaving between January and October 2013. Previous dysphagia training was described in the stroke care pathway and the community rehabilitation unit.

The most striking difference was the in-service training on the fractured neck of femur care pathway. Here, staff participated in the Surgical Services Directorate training programme, organised by 3 Education Leads. All the 800 RNs and support workers were rostered to attend 2 or 3 training days each year. The Education Leads asked to become trainers and then incorporated dysphagia into the 2013 training programme, thereby combining the mechanisms of hierarchical control and participatory adaption (Figs. 1, 2, 3 and 4).

There were differences in perceived staff knowledge about dysphagia. Clinical leaders in the stroke service spoke about their focus on dysphagia compared with other specialities. However, a change from plated meals, where the dietary requirements of patients are addressed centrally with the catering department, to serving breakfast from a trolley on the ward, where staff were required to select appropriate food textures for patients, proved challenging. A clinical leader observed: ‘It was huge concern-a real shock for us because we had a patient safety situation to deal with now’ (CL2). Participants from the fractured neck of femur care pathway reported limited dysphagia training. A trainer and support worker, who had been in post for more than 10 years, admitted ‘this was the first training I’d done … You just pick up what you see every day’ (T4).

### Outcomes and impact

Outcomes refer to specific references to the Inter-Professional Dysphagia Framework (IPDF) [22] in policy documents and the number of staff trained about dysphagia. Impact encompasses reported changes in practice on the 2 care pathways.

The IPDF was formalised in education policies and training programmes across the organisation. Awareness and Assistant Dysphagia Practitioner competence was added to the Preceptorship Policy and Essential Skills Log Book for newly qualified Registered Nurses in 2012; and reinforced in the corporate induction programme, which was attended by approximately 100 RNs in 2013. Awareness became part of the annual, mandatory training day for all occupational therapists and physiotherapists in 2013. The 2013 field notes record spread into the community, with 2 occupational therapists completing the train-the-trainer programme to roll out dysphagia training to rehabilitation assistants working in people’s homes in 2014 (Table 6). Awareness level knowledge featured in the education programmes for all support staff. An Education Strategic Lead said that between 250–300 support staff had completed dysphagia training over the last 3 years, commenting: ‘I think they’ve gained a lot … It’s sort of empowered them to talk to patients with some understanding about why it’s happening’ (ESL1). The IPDF also influenced staff development in the Surgical Services Directorate. Between January and October 2013, the Education Leads supported 176 RNs and 97 Clinical Support Workers to achieve Assistant Practitioner competence. They also negotiated an extra training course from the Speech and Language Therapy Department about dysphagia screening for 12 senior nurses, the next level of the Framework.

At a clinical level, most ward based trainers reported sharing their knowledge and skills informally as they cared for patients. Only 6 of the 25 trainers cascaded any dysphagia training. Increased awareness of dysphagia was the most frequently reported impact. Awareness, which included being more sensitive, applying knowledge and vigilance, was highlighted by trainers and verified by members of the multi-disciplinary team. A Clinical Lead noted that support staff’s ‘awareness of the different diets is very good now … I felt their knowledge is greater … They are more confident about observing people and what to observe’ (CL6). Several trainers described being more alert to risk factors, such as ‘I listen for people coughing’ (T4). There was a greater availability of, and appropriate use of specialist equipment. Five trainers spoke about obtaining adapted cutlery and crockery, thickener for drinks and shakers to mix drinks to the right consistency. One described how she and the ward manager tried to ban spouted beakers, because of the risk of choking, but ‘the cleaners would put them out again and some older patients would ask for them’ (T6) to avoid spillage.

## Discussion

This instrumental case study illustrates the particularity of planned change in context and over time. The spread and sustainability of a patient safety recommendation (using the Inter-Professional Dysphagia Framework [22] to structure workforce development) was tracked over 34 months in a large, health care organisation in England. The research aimed to explore the processes, mechanisms and outcomes of this locally developed innovation. Particularity, meaning the processes associated with a locally applicable change [34], was studied at an organisational and clinical level. Frameworks for spread [10] and sustainability [8] were blended to create a descriptive ‘small theory’ [26] expressly for the study. The ‘small theory’ specified the interventions (the mechanisms for spread); the processes associated with sustainability in organisations; the context and the expected outcomes. Using a ‘small theory’ added value by capturing the myriad, multi-level factors that influence spread and sustainability; and by offering clues to causality, as when hierarchical control and participatory adaptation worked in synergy to produce the desired outcomes, as on the fractured neck of femur care pathway.

Blending two frameworks provided an opportunity to move from the descriptive and conceptual, acknowledged limitations about work on spread [35]. The frameworks were selected because they are rooted in relevant literature. The spread mechanisms [10] had been used to categorise the transfer and implementation of Indigenous Australian health services and programmes [36]. The combined framework was logical, simple to explain and use. As far as we are aware, this is the first time these frameworks have been combined to underpin an empirical study.

A theory based approach was used a priori to optimise the accumulation of knowledge and to offer generalisable insights [37]. The case study shows the nuances of hierarchical control and participatory adaption, identifying features and processes associated with these mechanisms for the first time (see Figs. 1, 2, 3 and 4). Many of these features and processes replicate what is known about organisational and behaviour change. We were unable to test the third mechanism, facilitated evolution because the on-line version of the Dysphagia Toolkit was not available during the study period (see Additional file 1). However, it is questionable whether just creating the conditions for self-initiated change is appropriate, given that patient safety is not elective [38].

Hierarchical control was evident at the organisational, meso level, which is unsurprising as it is a top-down approach. Leadership and making the innovation routine practice were features of this mechanism (Figs. 1 and 2). Formal leaders agreed the policy and delegated delivery to middle managers, reflecting the view that leadership is a pre-condition for sustainability [12, 14]. Dysphagia education was formalised in policy and training programmes, thereby institutionalising the innovation [10, 17, 24, 39, 40]. In contrast, the three processes-critical junctures, the temporal dimension and stakeholder responses-were context specific, supporting the context dependent nature of change [41]. Critical junctures were linked with key stakeholders. Most decisive moments were associated with decision making meetings, a few were opportunistic and related to personal relationships and some did not materialise due to leadership inaction. The reasons were not explored, but may have been due to our failure to generate psychological buy-in [18]; whilst other leaders recognised the relative advantage of the innovation [42] from their clinical experience. Our findings reinforce that time is an essential aspect of organisational behaviour [41] and that change takes times [43]. The timeline for hierarchical control depicts a circular process starting with targeted dissemination, then negotiation, approval, allocation of resources, adaptation, implementation, maintenance support and ending with dissemination.

Participatory adaptation operated on the care pathways, the micro level, with the train-the-trainer intervention. As expected, the features and processes (Figs. 3 and 4) highlight particularity, given the path dependency of the processual perspective [24]. Only 6 of the 25 ward based Trainers delivered any dysphagia training. This finding mirrors what is known about train-the-trainer interventions: with reports of behaviour change among trainers but that the training may not be cascaded [18]. Trainers expressed their disappointment, attributing this to workload pressures. Resources are a common barrier to sustainability and spread [11, 12, 14, 16].

The expected outcomes were achieved when hierarchical control and participatory adaptation worked in tandem; when the top-down mandate was accompanied by engagement and support. The combined effect meant that hundreds of frontline staff received training about dysphagia for the first time at both organisational and clinical levels, suggesting a causal effect. This reflects top-down, bottom-up and push-pull approaches and supports the dynamic, context specific nature of spread and sustainability [13–15, 40, 44].

Participants reported specific, patient-focused indicators of impact. Trainers described how they applied and shared their new learning informally, as they cared for patients. They gave examples, such as obtaining adapted crockery and greater vigilance about coughing. This endorses a contingent approach with impact being context specific [11]. It is recommended that future research should evaluate patient related outcomes. This is difficult given the numerous confounding variables which influence patient outcomes, and the parlous state of evidence about the effectiveness of swallowing therapies for stroke and other impairments [5, 45–47]. There is an urgent need for well-controlled, adequately statistically powered treatment trials to evaluate the efficacy of dysphagia interventions.

Knowledge broking underpinned both mechanisms as the researchers were expected to span the boundaries between education, research and practice [32, 48]. The brokerage role was contextual [49] with a “directive and content-led style of facilitation” [50], p.viii that connected users with dysphagia knowledge. At the organisational level, the role involved negotiating with key stakeholders, spotting critical junctures and solving problems by for example, suggesting task and finish groups. On the care pathways, the role involved being proactive and reactive, adapting the out-reach support to the requirements of each setting (Table 2). The insider knowledge gained from the researchers being employees of the organisation facilitated collaboration and trust, important when dealing with such a value laden topic as patient safety.

A single site case study has many limitations, most notably whether the findings can be transferred to other settings. This was why an instrumental case study with an explicit, theory driven approach was used. The aim was to produce generalisable insights, by placing spread and sustainability at the forefront. This was supported by the prospective, longitudinal design and triangulating the findings from the different methods. We believe the findings provide more than a synoptic account of a specific innovation. They show the “fluidity, pervasiveness, open-endedness and indivisibility’ of change [41], p.456 in context over 34 months. The design meant we could explore temporality and confirm critical junctures. The contemporaneous field notes were important for tracking the processes and mechanisms; and the interviews were used to check the field notes. A limitation was the opportunistic sample of trainers, and more trainers participated in group than individual interviews. This was because their availability was dependent on gaining the permission of the ward manager during a busy shift. The knowledge broker role was a potential source of bias due to the researchers’ commitment to dysphagia as a patient safety issue. Bias was addressed through reflexivity, by acknowledging the dilemmas when moving between the ‘insider’ and ‘outsider’ researcher positions, through discussion and in the field notes [51].

The findings illustrate the duality of considering organisational change as context specific and universal. They support Jansson and colleagues’ factors that influence genuine, reciprocal knowledge transfer [43]. The context provided long-term institutional relationships, continuity of leadership, collaboration with regular face-to-face exchange and practical plans were negotiated with key stakeholders. There was a strong, institutional, multi-level partnership [32]; the researchers spanned boundaries acting as knowledge brokers [52]; and frontline personnel, the trainers were engaged as active change agents [18]. The researchers were involved with resource-intensive and strategic activities to keep dysphagia on the agenda. These efforts were helped by the natural links with nutrition and hydration, as fundamental aspects of care [53]. All these factors are both particular and ‘generalizable to theoretical propositions’ [54] p.15. Replicating our methodological approach across different settings, problems and interventions may illuminate these dualities and accelerate the development of an empirical base about spreading and sustaining healthcare innovations [55].

## Conclusion

This case study highlights the particularity of change, corroborates what is known about spread and sustainability and shows the value of theoretically informed research. Particularity is challenging, yet we would argue that the minutiae is both authentic and shows that implementation is ‘hard and slow’ [39], p.1. Two frameworks were blended, providing a theoretical lens to explore particularity. The processes associated with sustainability in organisations [8] were tracked in real time in a naturalistic context and combined with mechanisms for spread [10]. This blending offered new insights, identifying features and processes of the mechanisms of hierarchical control and participatory adaptation, and most importantly their synergist effect. These insights may be generalisable and warrant further investigation, especially in relation to patient safety, an area which requires constant attention to spread and sustainability.

## Additional files

Additional file 1: Description of the mechanisms for spreading the dysphagia recommendations. (DOCX 35 kb) Additional file 2: Interview topic guide. (DOCX 101 kb)

## Abbreviations

CL: Clinical lead
CLAHRC: Collaboration for Leadership in Applied Health Research and Care
CM: Catering manager
CSW: Clinical support worker
EL: Education lead
ESL: Education strategic lead
H: Housekeeper
IPDF: Inter-professional dysphagia framework
NHS: National Health Service
NIHR CLAHRC YH: National Institute for Health Research Collaboration for Leadership in Applied Health Research and Care for Yorkshire and Humber
OT: Occupational therapist
PSL: Professional strategic lead
RN: Registered nurse
SLT: Speech and language therapist
T: Trainer
