# Supplementary material for: Exploring factors that influence the spread and sustainability of a dysphagia innovation: an instrumental case study
Source: BMC Health Serv Res. 2016 Aug 18;16:406. doi: 10.1186/s12913-016-1653-6 (PMC4991017; doi:10.1186/s12913-016-1653-6)
Supplement: Additional file 1: — Description of the mechanisms for spreading the dysphagia recommendations. (DOCX 35 kb) [file 12913_2016_1653_MOESM1_ESM.docx]

**Description of the mechanisms for spreading the dysphagia recommendations**

***Hierarchical control***

**Definition:** A top-down push, planned and prescribed with decision made at a senior level with lower levels tasked and held accountable for making the change [1 p242].

**Description:** The timeline (April 2011 to January 2014) is shown in Tables 4 to 6. The features and processes associated with this mechanism are illustrated in Figures 1 and 2. The three processes are critical junctures, the temporal dimension and stakeholder response. The five features are endorsed by leaders, action delegated to middle managers, formalised in policy documents, fits into existing infrastructure, salient to patient safety care.

The study started in 2011 with targeted dissemination within the stroke unit and organisation to promote the uptake of the recommendations from the first study [2]. Between April 2011 and January 2012, the findings and recommendations were presented to over 100 multi-disciplinary staff working in hospital and community settings. They were discussed with committees with an organisation-wide remit for nutrition with patient representatives, and with nursing and allied health professional leaders and the stroke service.

Responsibility for producing an organisation-wide response, with an indication of priorities and resource requirements, was delegated to the Trust’s Nutrition Group. This committee formed a ‘task and finish group’ comprising senior managers representing education and speech and language therapy. A similar delegated approach was taken in the stroke service with a ‘task and finish group’ of multi-disciplinary front-line staff asked to develop a local action plan by managers. Both groups met twice and were facilitated by a researcher. The organisation-wide group reported back to the Nutrition Group, who in March 2012 ‘approved all the recommendations and the proposed action plan for the stakeholders.’ Such strategic level approval was a critical juncture.

Leadership support through delegating action to middle managers became evident over time. Examples were noted in the 2012 field notes and described by interviewees. One was a community unit response to a Professional Strategic Lead’s email which asked staff to gain Assistant Dysphagia Practitioner competence. In-house training sessions were organised by the speech and language therapist and about 25 multi-disciplinary staff from this unit gained the required competence. An Education Strategic Lead spoke about how she had been asked by the head of the department to ‘have an initial chat with the head of speech and language therapy about training housekeepers and domestics’ (ESL1). As a result, four education staff were trained to deliver dysphagia awareness, which was then incorporated into courses for new, and existing support staff across the organisation.

Hierarchical control was demonstrated through formalising the Inter-Professional Dysphagia Framework [3] in training policies, specifically the Essential Skills Log Book for newly qualified Registered Nurses (RNs) undergoing the local preceptorship programme [4]. This action was recommended by the education leader on the Nutrition Group and approved by nurse managers in July 2012. The researchers combined this mechanism with participatory adaptation, by offering support to the 80+ preceptors of the RNs. They were emailed to highlight the new standard and offered training resources. However, no preceptors (i.e. senior RNs who mentor newly qualified nurses) responded to this invitation. A master class about nutrition and dysphagia was introduced into the corporate induction for new qualified RNs. Five master classes, facilitated by the researchers, were attended by 85-100 RNs in 2013.

Interviewees most frequently referred to hierarchical control strategies for sustaining and spreading the focus on dysphagia. Twenty-five of the 30 interviewees suggested including dysphagia in induction, mandatory training, in-service training and preceptorship. A ward based Trainer said ‘I think in the mandatory training … when everyone starts at the hospital … It should be highlighted there … so everyone – doctors, nurses, clinical support workers, housekeepers … as many people as possible do the training in the induction’ (T5). A Strategic Education Lead considered that the ‘Nutrition and Hydration Accreditation Toolkit is a way to try to get things sustained: tools that clinical staff own but that are governed against’ (ESL2). The Toolkit was a local initiative to promote nutrition and allow regular monitoring of performance against specific criteria. We were unable to persuade colleagues that the Inter-Professional Dysphagia Framework should be included in the Hydration and Nutrition Assurance Toolkit.

***Participatory adaptation***

**Definition:** Engaging stakeholders in planning and delivering the change, through the provision of examples and support to promote local participation [1 p243].

**Description:** The features and processes associated with the train-the-trainer intervention are illustrated in Figures 3 and 4. The three processes are path dependency, the micro context and stakeholder response. The five features are on-going adaptation to fit a changing setting, patient need and staff knowledge, support and action by local leaders, workload pressures and competing priorities and resources available for immediate use.

Participatory adaptation comprised a train-the-trainer intervention to cascade dysphagia training on the care pathways for stroke and fractured neck of femur. The intervention lasted from January to October 2013 (see Table 6). It was multi-faceted, comprising a three hour session with a speech and language therapist (SLT), a Dysphagia Toolkit with teaching resources and information, three e-learning programmes which contained the essential knowledge, out-reach visits by the SLT, written and verbal feedback; and additional activities in response to requests (see Table 2). This responsive approach is the main reason for the variation in the number of outreach visits between the hospital and community rehabilitation unit. Secondary reasons were the availability of on-site speech and language therapy support in rehabilitation unit and that it was located several miles from the hospital sites.

A total of 28 staff completed the training: 25 were ward based staff and three were Education Leads from the Surgical Services Directorate. The ward based trainers were nominated by the ward manager, who was asked to identify front-line staff who assisted patients at mealtimes. The ward based Dysphagia Trainers included ten Registered Nurses (with four Ward Managers), 11 Clinical Support Workers, three House Keeping / Catering staff and one occupational therapist (see Table 3). The Education Leads were Registered Nurses.

The learning effect of the train-the-trainer intervention was evaluated using a postal survey, mealtime observations and the semi structured interviews.

*Survey:* The 2 page survey included demographic details, and six knowledge questions [5] and a dysphagia attitude scale [6]. It was distributed to all staff on the two care pathways before and after the intervention (see Tables 5-6). There was a response rate of 35.1% (91/255) on the pre intervention survey, and 35.7% (84/235) on the follow-up survey. The results showed an increase in knowledge post intervention, particularly for Registered Nurses, and an improvement in attitude amongst staff on the fractured neck of femur care pathway.

*Structured mealtime observations:* A bespoke mealtime observation schedule was developed to assess staff behaviour pre and post the train-the-trainers intervention. The observation schedule recorded staff adherence to patient specific Speech and Language Therapy swallowing recommendations recorded on yellow swallowing status sheets. The observations on the care pathways were done by the speech and language therapist/researcher who had delivered the training. A total of 19 meals with 33 staff-patient interactions were observed before the intervention and, 21 meals with 30 staff-patient interactions after the intervention. Overall, there was an improvement in the percentage of staff looking at the yellow sheets (54.2% Pre/76.2% Post*), correct positioning of patients (86.2% Pre/88.9% Post) and adherence to specific advice (53% Pre/77.8% Post); and a decrease in the recommended level of supervision/assistance (91.3% Pre/82.6% Post*), the correct equipment being used (100% Pre/80% Post*) and patients sat upright for 30 minutes after the meal (78.9% Pre/70% Post).

*Interviews:* All 12 trainers were positive about the training with several describing it as ‘eye-opening.’ The most memorable topics related to theory and practice. ‘It’s made me more aware of why you have to thicken fluids. There is a reason behind … understanding why they have different levels of thickness’ (T8). Also, the swallowing process: ‘seeing how a swallow goes and comparing a normal swallow with someone who’s got dysphagia’ (T1). The practical session was highlighted too. For example ‘adding too much thickener and leaving it to become solid’ (T6) and being ‘in the position where we fed each other. You realise ‘oh god it’s not nice - how that patient feels, how uncomfortable’ (T1).

Trainers were encouraged to negotiate a training plan with their ward manager and to adapt the dysphagia training to their setting. The field notes about the outreach visits to support the ward Trainers in their new role, show that most were ‘struggling’ to start cascading the training within the first month. This was because of staff shortages and clinical priorities. Dysphagia training started on one post-operative ward. The trainers negotiated dedicated time (a 20 minute slot when the two staff on two separate shifts overlapped) with the ward manager, who had also completed the training, and organised a training rota. However, this training stopped when the protected time was withdrawn due to workload pressures ‘we didn’t have time to fit everyone in’ especially with the problems of off-duty rostering’ (T7). The difficulties delivering training was raised as part of the feedback with the clinical leaders at the mid-way and final meetings (Table 2), verbally and in the reports.

After six months, six ward based Trainers had supported an estimated total of 37-47 colleagues to achieve Awareness and Assistant Dysphagia Practitioner competence. This was mainly in the stroke unit and a post-operative ward (see Table 2). The trainers and clinical leaders offered several reasons, including low staffing levels, workload pressures, patient related priorities, lack of time and rostering which meant it was difficult for staff to be released for training at the same time as the Trainer. One expressed her disappointment: ‘I selected two people, and they both agreed but we’ve never been on at the same time …so I am really sorry about that’ (T3).

The three Education Leads, who volunteered to become Trainers, incorporated the Dysphagia Framework in the 2013 training programme for the Surgical Services Directorate.

All the 800 Registered Nurses and support workers in the Directorate were rostered to attend two or three training days, each year. Each trainer described adapting the Dysphagia Toolkit. For example, ‘in the practical session, rather than delivering it as a PowerPoint presentation, they read the printed PowerPoint … and are directed to certain activities – worksheets that came with the toolkit, and then they have a go at thickening the fluids … Sometimes I turn it into a competition – the group that get it right, receive a free cup of tea’ (EL&T2). The Education Leads combined hierarchical control and participatory adaption.

***Facilitated evolution***

**Definition:** Creating the conditions and capability for defining problems, initiating problem solving and action, with accountability for the change [1 p243].

**Description:** The third intervention –putting the Dysphagia Toolkit on the organisation’s intranet for staff to use - did not take place during the research. The research team agreed to defer this intervention until the Toolkit had been evaluated by the trainers and eight health care professionals from three organisations. This meant that the transfer to an electronic format was delayed until 2014 when it was published on the internet, see http://www.dysphagiatoolkit.nihr.ac.uk/ [7]. It was not possible to monitor its uptake as part of the research study.

**References**

1. Ovretveit J. Widespread focused improvement: lessons from international health for spreading specific improvements to health services in high-income countries. International Journal of Quality in Health Care. 2011; 23: 239-246 doi:10.1093/intqhc/mzr018

2. Ilott I, Bennett B, Gerrish K, Pownall S, Jones A, Garth A. Evaluating a novel approach to enhancing dysphagia management: workplace based, blended e-learning. Journal of Clinical Nursing. 2014; 23:1354-1364 doi: 10.1111/jocn.12409.

3. Boaden E, Davies S, Storey L, Watkins C. Inter-professional Dysphagia Framework. 2006. [http://www.dysphagiatoolkit.nihr.ac.uk/background-to-the-south-yorkshire-dysphagia-toolkit/The-Inter-Professional-Dysphagia-Framework. Accessed 20 April 2016](http://www.dysphagiatoolkit.nihr.ac.uk/background-to-the-south-yorkshire-dysphagia-toolkit/The-Inter-Professional-Dysphagia-Framework.%20Accessed%2020%20April%202016).

4. Department of Health. Preceptorship framework for newly registered nurses, midwives and allied health professionals. 2010 <http://webarchive.nationalarchives.gov.uk/20130107105354/http://www.dh.gov.uk/prod_consum_dh/groups/dh_digitalassets/@dh/@en/@abous/documents/digitalasset/dh_114116.pdf> Accessed 23 April 2016.

5. Payne D. Dysphagia Quiz. 1994. Published as part of the training pack ‘Managing Dysphagia’ produced by Winchester and Eastleigh Healthcare Trust.

6. Colodny, N. Construction and validation of the Mealtime and Dysphagia Questionnaire: An Instrument Designed to Assess Nursing Staff Reasons for Noncompliance with SLP Dysphagia and Feeding Recommendations. *Dysphagia*, 2001; 16, 263-271.

7. Eltringham S, Pownall S, Ilott I, Bennett B & Taylor C. South Yorkshire Dysphagia Toolkit. 2013. ISBN 978-1-908492-06-7 Available at <http://www.dysphagiatoolkit.nihr.ac.uk/> Accessed on 22 April 2016.
