# Supplementary material for: Exploring factors that influence the spread and sustainability of a dysphagia innovation: an instrumental case study
Source: BMC Health Serv Res. 2016 Aug 18;16:406. doi: 10.1186/s12913-016-1653-6 (PMC4991017; doi:10.1186/s12913-016-1653-6)
Supplement: Additional file 2: — Interview topic guide. (DOCX 101 kb) [file 12913_2016_1653_MOESM2_ESM.docx]

**Exploring scale-up, spread and sustainability:**

**tracing a health care innovation about dysphagia**

**Interview topic guide**

**Interview topics and probes**

**1. Background:** designation and tenure, role of participant in relation to the dysphagia project.

**2. Dysphagia recommendations:** how and when became aware of the project; understanding of the recommendations for the Trust, and if appropriate the stroke / fractured neck of femur care pathway.

**3. Perceptions of the recommendations:** areas of agreement and disagreement; what’s missing; views about the feasibility and desirability about implementation.

**4. Implementation:** observations about if, how, where, which of the recommendations have been implemented or not; in the Trust and by multi-disciplinary teams in clinical areas; any adaptations with reasons for tailoring the recommendations.

**5. Barriers and facilitators to implementation:** identification of what has helped and hindered the uptake of the recommendations; influence of competing priorities, personal relationships, professional networks and resources.

**6. Actions supporting implementation:** comments about dissemination; formalisation in policy documents; the ‘training the trainer’ intervention; any other mechanisms; views of the effectiveness of each action and the inter-action between them.

**7. Changes in dysphagia management:** observations/evidence of expected and unexpected differences in clinical practice; influence of any other, on-going activities/initiatives.

**8. Sustainability and spread:** observations/evidence of change in practice being maintained and spread in expected and unexpected ways; identifying the mechanisms, key agents and reasons; views about the longer-term prospects for maintaining the change/innovation.

**9. Any other questions/comments?**
